# Optimizing Conditions for Microwave-Assisted Extraction of Polyphenolic Content and Antioxidant Activity of *Barleria lupulina* Lindl.

**DOI:** 10.3390/plants10040682

**Published:** 2021-04-01

**Authors:** Noor Wahida Ismail-Suhaimy, Siti Salwa Abd Gani, Uswatun Hasanah Zaidan, Mohd Izuan Effendi Halmi, Paiman Bawon

**Affiliations:** 1 Halal Products Research Institute, Universiti Putra Malaysia, Putra Infoport, Serdang 43400, Selangor, Malaysia; noorwahidaismailsuhaimy@gmail.com; 2 Department of Agriculture Technology, Faculty of Agriculture, Universiti Putra Malaysia, Serdang 43400, Selangor, Malaysia; 3 Department of Biochemistry, Faculty of Biotechnology and Biomolecular Sciences, Universiti Putra Malaysia, Serdang 43400, Selangor, Malaysia; uswatun@upm.edu.my; 4 Department of Land Management, Faculty of Agriculture, Universiti Putra Malaysia, Serdang 43400, Selangor, Malaysia; m_izuaneffendi@upm.edu.my; 5 Department of Forest Production, Faculty of Forestry, Universiti Putra Malaysia, Serdang 43400, Selangor, Malaysia; paiman@upm.edu.my

**Keywords:** *Barleria lupulina* Lindl., microwave-assisted extraction (MAE), response surface methodology (RSM), total flavonoid content (TFC), total phenolic content (TPC), 1-diphenyl-2-picrylhydrazyl (DPPH), 2,20-azino-bis (3-ethylbenzothizoline-6-sulfonic acid) (ABTS)

## Abstract

*Barleria lupulina* Lindl. (Acanthaceae) as an ornamental plant has been widely used in folklore medicine due to its abundancy in polyphenolic compounds. The present study examined conditions for optimal extraction of antioxidants from *B. lupulina* leaf extracts by using the microwave-assisted extraction (MAE) method. The effects of ethanol concentrations, microwave power, and extraction time on total phenolic content (TPC), total flavonoid content (TFC), 1-diphenyl-2-picrylhydrazyl (DPPH), and 2,20-azino-bis (3-ethylbenzothizoline-6-sulfonic acid) (ABTS) were investigated by single-factor experiments. Response surface methodology (RSM) was applied to observe interactions of three independent variables (ethanol concentrations, microwave power, and extraction time) on the dependent variables (TPC, TFC, DPPH, and ABTS) to establish optimal extraction conditions. Quadratic polynomial equations in all experimental models yielded favorably with fitted models with R^2^ and R^2^_adj_ of more than 0.90 and a non-significant lack of fit at *p* > 0.05. The optimal conditions for the extraction of antioxidant activity were established at 80% (*v/v*) ethanol, 400 W, and 30 s with TPC (238.71 mg gallic acid equivalent (GAE)/g sample), TFC (58.09 mg QE/g sample), DPPH (87.95%), and ABTS (89.56%). Analysis by ultra-high-performance liquid chromatography–quadrupole time-of-flight mass spectrometry (UHPLC-QTOF/MS) successfully identified four new phenylethanoid glycoside compounds in the species.

## 1. Introduction

Polyphenols are a group of phytochemicals produced by plants as secondary metabolites. These compounds are known to give health benefits to humans, including anti-aging, anti-tumor, anti-carcinogenic, and anti-glaucoma properties [1]. They are known to be the most prevalent antioxidants in plants and allegedly possess both the capacity to quench oxygen radicals and inhibit the development of free radicals [2]. Flavonoids are a family of polyphenols with subclasses, including flavones, flavanols, isoflavones, flavanones, and chalcones. They are known to be highly effective antioxidants and possess anti-inflammatory, antimicrobial, antiviral, anti-mutagenic, and anti-carcinogenic properties [3,4,5]. Flavonoids are known to be essential components in a number of nutraceutical, pharmaceutical, medicinal, and cosmetic products [3]. The increasing awareness of the health benefits associated with the use of polyphenols has led to the expanding demand in the food, beverage, pharmaceutical, and cosmetics industries [6].

*Barleria lupulina* Lindl. is a pantropical herb and is widely cultivated as an ornamental shrub. The species belongs to the Acanthaceae family that comprises 300 species [7]. It is commonly known as the *hophead philippine* violet and by various local names, such as Penawar seribu bisa (Malaysia) [8], Landik (Indonesia) [9], Sa-let-pangpon, Chong-ra-ar (Thailand) [10], Kanta vishalyakarni (India), and Neel saireyak (Sanskrit) [11]. Traditionally, *B. lupulina* was used to treat several diseases, including treating snake bites, dog bites, swelling, bleeding wounds, rheumatism, herpes simplex, and herpes zoster [10,11]. Studies have documented that *B. lupulina* has medicinal values, including anti-inflammation [12], antiviral [13,14], antibacterial [11,15,16], antidiabetic [17], cytotoxic [18,19], antidiuretic, and antiarthritic activity [20,21]. Several studies have also shown that the species possesses highly potential antioxidants and bioactive compounds used in treatments of various diseases and health issues [10,15,19,22,23].

One important consideration in the extraction of bioactive compounds from plant materials is the preparation of the extract and the extraction procedures [24]. Studies have shown that there is a strong relationship between type and polarity of extraction solvents, time, temperature, physical characteristics of samples, and amount of polyphenols extracted [25]. Heating is known to influence the content of some polyphenols by the rupturing of the cell membrane, causing the release of membrane-bound phytochemicals and the increase in bioavailability [26]. Several studies suggested that recent sample preparation and extraction procedures are superior in overcoming the disadvantages of traditional methods and, thus, are able to increase efficiency of extraction by using less consumption of solvents and time of extraction [27,28,29,30]. Microwave-assisted extraction (MAE) is among the more recent procedures, which involves the heating up of molecules by a dual mechanism of ionic conduction and dipole rotation. The procedure involves the disruption of cell walls and the release of compounds of interest to the extracting solvent [31]. The advantage of MAE is its shorter extraction time, as well as its stability and reproducibility [32,33].

Response surface methodology (RSM) is software used for analyzing and optimizing data based on mathematical and statistical models. It is widely used in experimental designs for the optimization of experiments [34]. RSM has the capability to scrutinize and summarize experimental parameters (dependent variables), responses (independent variables), and their interactions, hence saving time, cost, and the amount of materials used [35]. RSM has been widely used in several fields, such as industrial crops and products [36], food chemistry [29], and renewable energy [37,38]. Previous studies have shown that RSM was useful in establishing optimal conditions for the extraction of antioxidant compounds and their activities [39,40,41].

The MAE is an efficient method due to its ability to extract bioactive compounds from *B. lupulina* leaves in a shorter time period as compared to Soxhlet and ultrasound-assisted extraction (UAE) [42]. The present study investigated the effects of solvent concentrations, extraction time, and microwave power on extraction efficiency using MAE procedures conducted in single-factor experiments. RSM was utilized to establish the optimal conditions preceding the ultra-high performance liquid chromatography–quadrupole time-of-flight mass spectrometry (UHPLC-QTOF/MS) to determine the bioactive compounds.

## 2. Results and Discussion

### 2.1. Analyses of Single Factors

#### 2.1.1. Effects of Ethanol Concentrations

The choice of extraction solvent generally affected the amount and types of extracted compounds as cited by [43]. Ethanol has several advantages over other solvents, such as higher extraction ability, environmental compatibility, lower toxicity, and cost, and it is the most commonly used solvent in the extraction of phytochemicals from plant extracts [30,43]. Ethanol concentrations in water have been reported to have significant effects on plant extraction quality [44,45]. In the present study, *B. lupulina* Lindl. leaf extract (BLLE) was investigated at five levels of ethanol concentrations (20, 40, 60, 80, and 95%, *v/v*) while keeping other conditions (1000 W and 60 s) constant.

**Figure 1:**
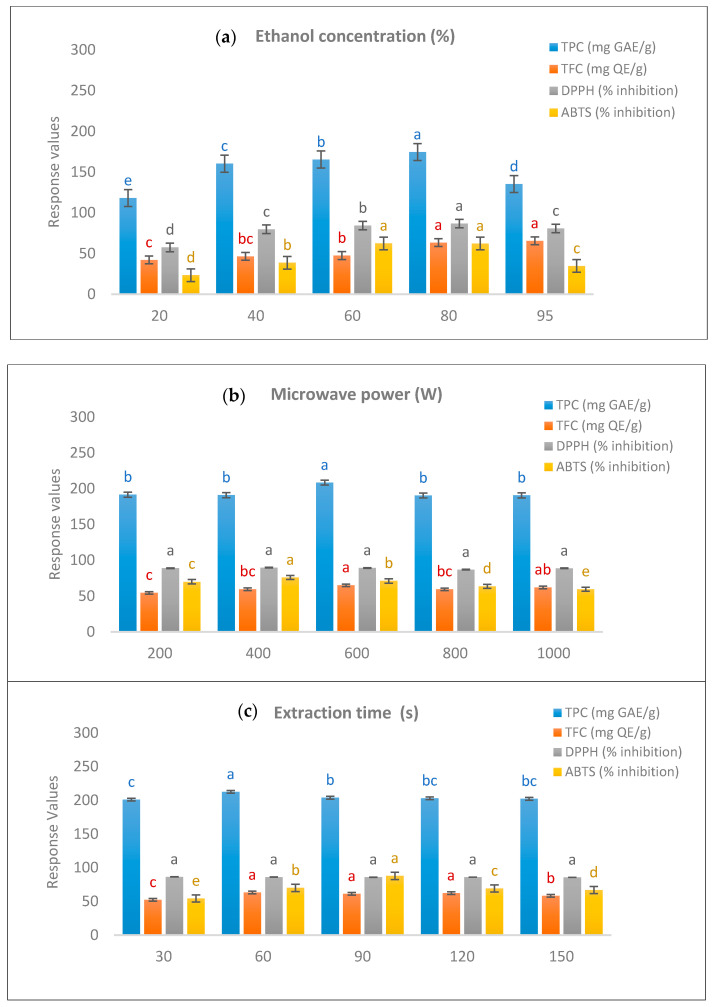
Effects of single factors: (**a**) ethanol concentrations, (**b**) microwave power, and (**c**) extraction time on total phenolic content (TPC), total flavonoid content (TFC), 1-diphenyl-2-picrylhydrazyl (DPPH), and 2,20-azino-bis (3-ethylbenzothizoline-6-sulfonic acid) (ABTS), respectively, of B. lupulina leaf extract. One-way ANOVA was used to compare the significant differences among groups. Different letters (a, b, c, d, and e) in blue, red, black, and yellow colors, respectively, represent significant differences (*p* < 0.05) in TPC, TFC, DPPH, and ABTS values among groups. The same letter indicates no significant difference (*p* > 0.05) among groups.

Figure 1a shows an increase in total phenolic content (TPC) when extracting at 20–80% (*v/v*) ethanol concentrations but a decrease at 95%. In contrast, increase in ethanol concentrations yielded increases in the yield of total flavonoid content (TFC). The 1-diphenyl-2-picrylhydrazyl (DPPH) and 2,20-azino-bis (3-ethylbenzothizoline-6-sulfonic acid) (ABTS) showed better scavenging activity at 60–80% (*v/v*) ethanol concentrations. Ethanol concentrations of between 20 and 80% (*v/v*) were selected for RSM, and an 80% concentration was used in the subsequent experiments. 

#### 2.1.2. Effects of Microwave Power

Microwave power is an important factor in microwave-assisted extraction procedures. It is known that the efficiency of extraction of phytochemicals depends on the volumetric heating of plant cells. At the parameters of 80% (*v/v*) and 60 s, the influence of microwave power at 200, 400, 600, 800, and 1000 W was measured.

Figure 1b presents the parabolic effects of microwave power on TPC and TFC, both attaining peaks at 600 W. Increase in microwave power could increase extraction efficiency through maximizing molecular interactions between the electromagnetic field and the sample as reported by [46]. However, prolonged exposure to a higher microwave power could degrade some phenolic compounds [47]. In the present study, percentage inhibition of scavenging activity showed that 400 W was better for ABTS activity but had no significant difference in DPPH activity. The 600 W microwave power was considered proper for the present subsequent experiments.

#### 2.1.3. Effects of Extraction Time

Extraction time in a plant extraction procedure is considered very important, as it could result in saving time and cost. Based on the previous experiments in the present study, extraction efficiency was measured at extraction times of 30–150 s at the optimized extraction conditions of 600 W and 80% (*v/v*). 

Data in Figure 1c show that TPC yielded its highest level at a 60 s extraction time compared with other durations. There was no significant difference in TFC at extraction times of between 60 and 120 s. In scavenging activity, DPPH showed no significant difference between times of extraction, while the highest activity for ABTS scavenging was recorded at 90 s, which was higher than that of the others. A longer extraction time is known to degrade the antioxidants in the extract.

### 2.2. Analysis of Response Surface Methodology (RSM) 

#### Fitting Model

Analysis of variance (ANOVA) was performed to determine the quadratic model of the experiments. The significance of the model was determined by a high F-value and a low *p*-value (<0.5) [48]. The reliability of the model was determined by a lack of fit value, where a *p*-value of more than 0.05 was not significant. The coefficient of determination (R^2^) of the model referred to the correlation between predicted and experimental data, where the lesser the difference between R^2^ and adjusted R^2^, the better the statistical model.

Table 1 presents responses for each model showing its significance where all *p*-values were less than 0.05. High significance was shown in DPPH (F-value = 350.98; *p* < 0.0001) and ABTS (F-value = 29.12; *p* < 0.0001), followed by TFC (F-value = 23.52; *p* < 0.0002) and TPC (F-value = 20.17; *p* < 0.0003). The lack of fit value for each response showed that the model was valid and fitted well with a value greater than 0.05 (not significant). TPC, TFC, DPPH, and ABTS gave values of 0.7414, 0.8221, 0.5152, and 0.49447, respectively, suggesting that all of the models had significant effects on parameters of output responses [49]. All responses showed R^2^ and adjusted R^2^ of less than 0.2, which were not significantly different. The results indicate that the statistical models were good.

### 2.3. Conditions for Optimal Extraction

#### 2.3.1. Total Phenolic Content

The total phenolic content (TPC) from *B. lupulina* Lindl. leaf extract using the MAE method ranged from 149 to 239.33 mg GAE/g with a mean of 209.06 mg GAE/g. The highest yield in TPC (239.33 mg GAE/g) was observed in experimental run no. 7 under extraction conditions of 80% (*v/v*) ethanol concentration, 400 W microwave power, and an extraction time of 30 s. The extraction procedures in the present study yielded higher extraction values when compared to previous maceration [18] and Soxhlet methods [50].

The ANOVA of regression coefficient showed a linear response based on the *p*-value of ethanol concentrations, which were highly significant (*p* < 0.0001), followed by microwave power and extraction time (Table 2). In the quadratic model, only ethanol concentrations (A^2^) were significant (*p* < 0.05). The interaction between variables, ethanol concentration × power (AB), and power × extraction time (BC) had a significant impact on TPC (*p* < 0.05). 

**Figure 2:**
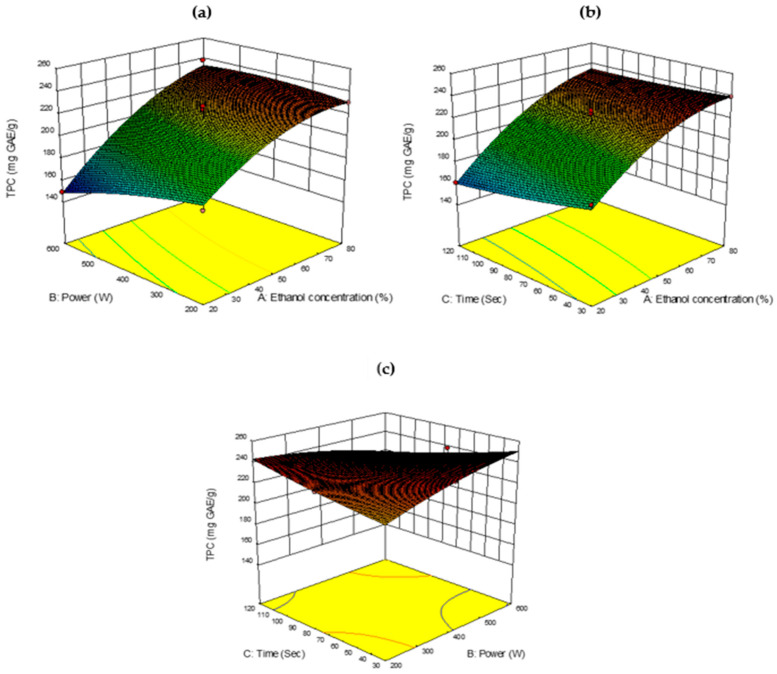
Response surface analysis (3D) of *Barleria lupulina* Lindl. leaf extract (BLLE) on total phenolic content (TPC): (**a**) effect of ethanol concentration and power; (**b**) effect of ethanol concentration and time; (**c**) effect of power and time.

In the present experiment, as the ethanol concentration increased from 20 to 80% (*v/v*), the yield of TPC in the extract increased, as shown in Figure 2a. Ethanol concentration also showed significance in quadratic and interactions with microwave power, and non-significance (*p* > 0.05) in interaction time. The ability of the mixture in any proportion between water (strong polar solvent) to ethanol (low polar solvent) was cited to increase polarity of the complex solvent [51]. In the present study, 80% (*v/v*) ethanol was a good proportion to obtain a high TPC yield. With respect to microwave power, TPC reached its maximum at 400 W and slightly dropped with an increase in microwave power (Figure 2b). This could be due to the thermal degradation of phytochemicals at higher microwave power levels. The higher heat generated by higher microwave power with volumetric heating could be too strong for plant cells, causing the breakdown of phytochemicals [29]. 

In the present study, 400 W was considered a better option to obtain an optimal yield. Extraction time did not influence the yield of TPC (Figure 2c). In general, the quantity of analytes that can be extracted from a sample has been reported to improve by increasing the extraction time. However, there was a chance that extracted compounds could be degraded [29]. It was observed that 30 s of irradiation time in the present study was able to obtain a better yield of TPC, in agreement with findings by [52].

#### 2.3.2. Total Flavonoid Content

The total flavonoid content (TFC) from *B. lupulina* Lindl. leaf extract using MAE ranged from 29.1527 to 65.2672 mg QE/g with a mean value of 45.2357 mg QE/g. The highest TFC yield (65.2672 mg QE/g) was observed in experimental run no. 8 under extraction conditions of 80% (*v/v*) ethanol concentration, 400W microwave power, and an extraction time of 120 s. The ANOVA of the regression coefficient indicated that the two linear parameters, ethanol concentrations (A) and microwave power (B), were significant at *p* < 0.0001 and *p* < 0.05, respectively (Table 2). The quadratic (A^2^) and interaction effects between ethanol concentrations and extraction time (AC) were also significant (*p* < 0.05) on TFC yield. 

**Figure 3:**
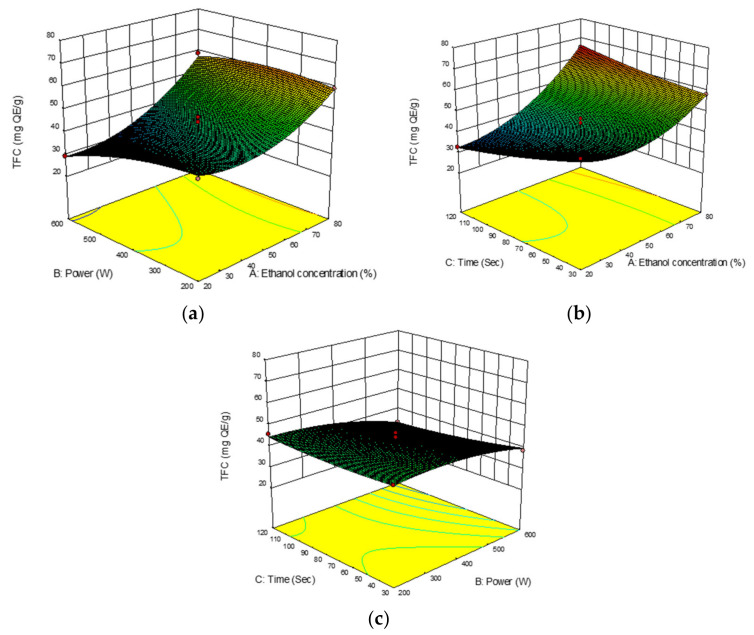
Response surface analysis (3D) of *Barleria lupulina* Lindl. leaf extract (BLLE) on total flavonoid content (TFC): (**a**) effect of ethanol concentration and microwave power; (**b**) effect of ethanol concentrations and extraction time; (**c**) effect of microwave power and extraction time.

Heat generated by microwave power generally caused interactions between the sample and solvent, which caused disruption of the sample to occur and the release of analyte in the solvent [53,54]. It was reported that an increase in the heat produced increase in the yield of the extract [55] and at a faster rate [53]. Heating could also cause an increase in solubility of flavonoids from the plant matrix by disrupting the phenolic matrix bonds [56]. Figure 3a shows the interactions between ethanol concentrations and microwave power on the yield of TFC. An increase in microwave power from 200 to 400 W in the present study caused an increase in TFC yield but caused a decrease beyond 400 W. This could be the degradation of thermolabile compounds due to the heat produced [54,57]. Figure 3b shows an increase in the value of TFC with an increase in ethanol concentrations and extraction time. The positive interaction of solvent concentration and time could enhance solubility of TFC at a minimum level of heating power, which suggests it as an alternative way to avoid the degradation of compounds. However, the interaction of time and microwave power showed a negative effect on TFC yield (Figure 3c). The increase in extraction time and microwave power caused a decrease in TFC yield. This could be related to the high dielectric properties of ethanol as a polar solvent, and combining it with water could cause an increase in the rate of heating that tends to degrade the compounds over a prolonged exposure to microwave power.

#### 2.3.3. DPPH Activity

**Figure 4:**
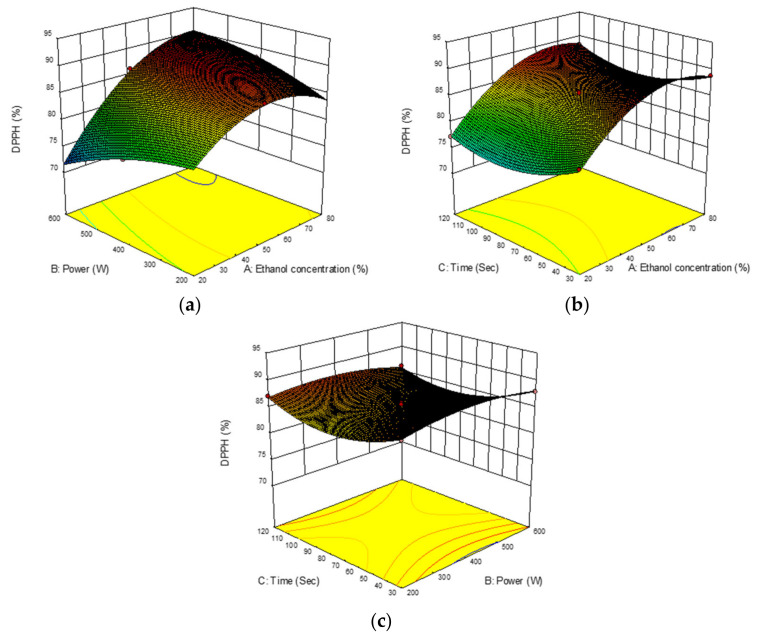
Response surface analysis (3D) of *Barleria lupulina* Lindl. leaf extract (BLLE) on DPPH activity: (**a**) effect of ethanol concentrations and microwave power; (**b**) effect of ethanol concentrations and extraction time; (**c**) effect of microwave power and extraction time.

In the present study, antioxidant activity of *B. lupulina* Lindl. leaf extract towards DPPH radical assay ranged from 70.21 to 88.75% with a mean of 83.91%. The highest DPPH activity (88.75%) was recorded in experimental run no. 7 under extraction conditions of 80% (*v/v*) ethanol concentration, 400 W microwave power, and an extraction time of 30 s. The ANOVA revealed that only ethanol concentrations (A) had a positive significant linear effect (*p* < 0.0001) (Table 2). The increase in ethanol concentrations and microwave power caused a significant decrease in DPPH activity (negative quadratic effect, A^2^ and B^2^) in contrast with extraction times (C^2^), which caused significant increases in DPPH activity (*p* < 0.0001). Positive significant interaction effects (*p* < 0.0001) were recorded between ethanol concentrations with microwave power (AB) and ethanol concentrations with extraction times (*p* < 0.05) (AC). Antioxidant capacity of DPPH was observed to increase when ethanol concentrations were increased at a minimum microwave power between 200 and 400 W (Figure 4a) with an increase in extraction times (Figure 4b). Meanwhile, negative interaction effects between extraction times and microwave power (BC) showed an increase in antioxidant capacity of DPPH with prolonged extraction times but at a minimum microwave power of between 300 and 400 W (Figure 4c).

The DPPH radical scavenging assay has been widely used because of its stabilization of free radicals—a simple, rapid, and convenient method for estimating antiradical activity. The assay measures the ability of a substance, or a complex mixture of substances (antioxidants), to scavenge free radicals through donation hydrogen atoms or electrons. The reaction of a hydrogen-donating antioxidant can be seen by the changes of the purple alcoholic DPPH solution to green and yellow. The DPPH assay has been quoted to be more favorable to react with low molecular weight phenolic compounds [58]. Based on observations made in the DPPH assay, TPC yielded more than 180 mg GAE/g and showed more than 80% DPPH activity. The effects of ethanol concentrations of between 20 and 80% *(v/v*) in the DPPH assay in the present study mirrored previous studies on *Orthosiphon stamineus* extracts, which were related to the highly active and moderately polar phenolic compounds [45]. DPPH activity initially yielded high activity but decreased upon reaching a minimum extraction time of between 70 and 80 s, before improving activity. The results were in agreement with the findings of other studies in which DPPH scavenging capacity increases with an increase in extraction time [36,59].

#### 2.3.4. ABTS Activity

**Figure 5:**
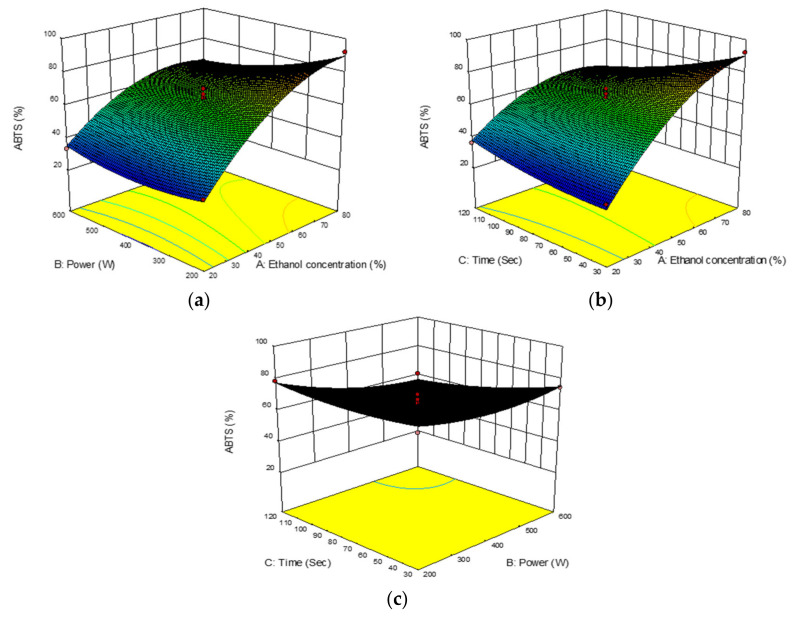
Response surface analysis (3D) for *Barleria lupulina* Lindl. leaf extract (BLLE) on ABTS activity: (**a**) effect of ethanol concentrations and microwave power; (**b**) effect of ethanol concentrations and extraction time; (**c**) effect of microwave power and extraction time.

ABTS activity on *B. lupulina* Lindl. leaf extract ranged from 30.08 to 92.08% with a mean of 61.75%. The highest ABTS yield (92.082%) was observed in experimental run no. 7 under extraction conditions of 80% (*v*/*v*) ethanol concentration, 400 W microwave power, and an extraction time of 30 s. The ANOVA showed that only ethanol had positive significant linear effects (*p* < 0.0001) (Table 2) when compared to microwave power (B) and extraction time (C), with ABTS activity being significantly decreased (linear negative effect) (*p* < 0.05). The negative interactions between the variables were seen as being non-parallel between variables. ABTS activity increased with increasing ethanol concentrations indicating it to be more effective at a lower microwave power (AB) (Figure 5a) and lower time of extraction (AC) (Figure 5b).

The results were in agreement with the findings of other studies in which the polarity of solvent used was possibly due to solubility of phenolic compounds responsible for antioxidant activities [41]. Similar with other findings of the interaction between microwave power and extraction time, the ABTS activity decreased with an increase in microwave power and prolonged time of extraction (BC) (Figure 5c). These conditions could be related with the absence or denaturing of bioactive compounds, which had the potential to scavenge because of the exposure to high heat in the longer extraction time. The extract with high ABTS activity was correlated with high values of TPC, indicating a correlation between antioxidant activity and polyphenols content. The occurrence was probably due to the polarity of the solvent used, which coincided with the solubility of the phenolic compounds responsible for ABTS activity.

In the analyses, the second-order model equation (Equations (1)–(4)) could be used to predict the responses:

Y*_TPC_*= 140.92 + 1.72*A* + 0.07*B* + 0.16*C* + 1.59(10^−3^)*AB* + 3.27(10^−3^)*AC* − 1.48(10^−3^)*BC* − 0.02*A^2^* − 9.34(10^−5^)*B^2^* + 8.08(10^−4^)*C^2^* (1)

Y*_TFC_*= 69.89 − 1.06*A* + 0.03*B* − 0.26*C* + 5.28(10^−^^4^)*AB* + 4.38(10^−^^3^)*AC* − 1.91(10^−^^4^)*BC* + 8.84(10^−^^3^)*A^2^* − 7.37(10^−^^5^)*B^2^* + 5.38(10^−^^4^)*C^2^* (2)

Y*_DPPH_*= 81.16 + 0.41*A* − 1.04(10^−^^3^)*B* − 0.25*C* + 6.37(10^−^^4^)*AB* + 4.83(10^−^^4^)*AC* − 4.64(10^−^^5^)*BC* − 5.39(10^−^^3^)*A^2^* − 3.48(10^−^^5^)*B^2^* + 1.51(10^−^^3^)*C^2^* (3)

Y*_ABTS_*= −26.42 + 3.12*A* − 0.03*B* + 0.26*C* − 1.08(10^−^^3^)*AB* − 7.21(10^−3^)*AC* − 5.24(10^−4^)*BC* − 0.01*A^2^* + 1.17(10^−4^)*B^2^* + 1.44(10^−3^)*C^2^* (4)

Y indicates predicting responses, and A, B, and C represents ethanol concentrations, microwave power, and extraction time, respectively.

### 2.4. Verification of Model

A set of conditions were provided to verify the model. Desirability function was used to optimize the conditions by choosing a minimum for the time of extraction and in range for ethanol concentrations and microwave power. The conditions purposed for *Barleria lupulina* Lindl. leaf extract included 80% (*v/v*) ethanol concentration, 400 W, and 30 s at a desirability of 95.1%. Data were statistically compared between the suggested extraction conditions with the predicted values given by RSM software. The results show close agreement at a 95.1% confidence level between verification experiments with predicted values, as shown in Table 3.

### 2.5. Identification of Phytochemical Compound in B. lupulina Lindl.

**Figure 6:**
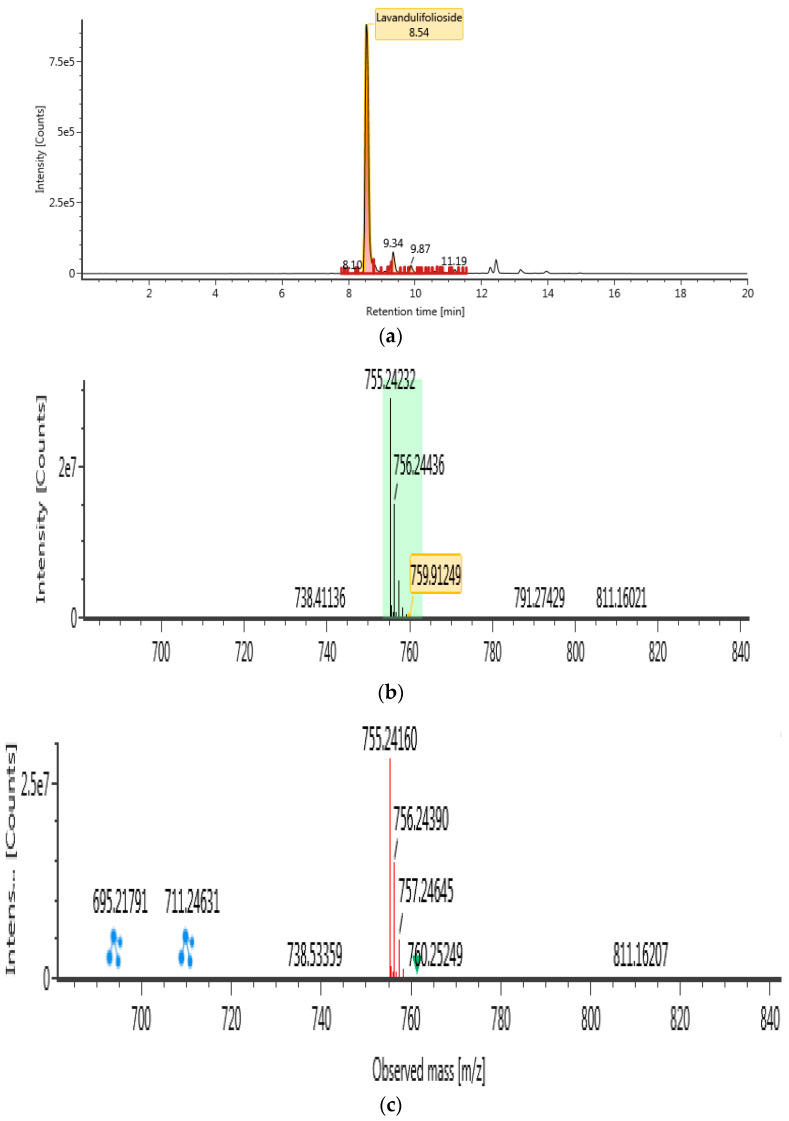
UHPLC-QTOF/MS chromatograms of lavandulifolioside from *Barleria lupulina* Lindl. extract (BLLE): (**a**) chromatogram mass spectrum; (**b**) low energy of mass spectrum; (**c**) high energy of mass spectrum.

**Figure 7:**
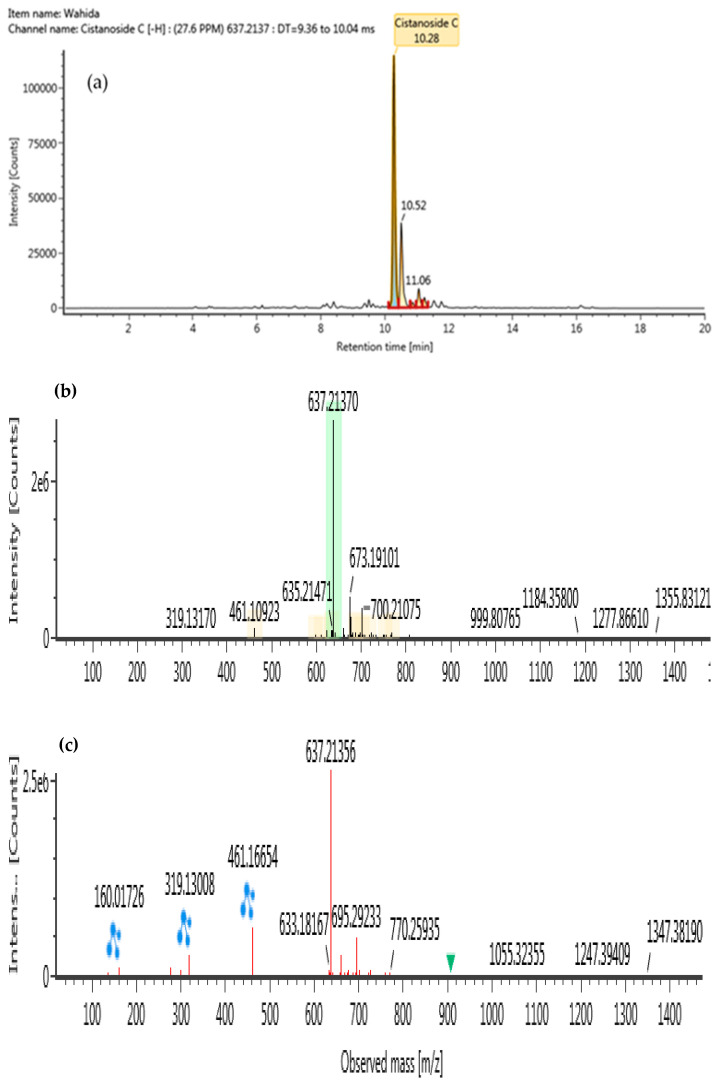
UHPLC-QTOF/MS chromatograms of cistanoside C from *Barleria lupulina* Lindl. leaf extract (BLLE): (**a**) chromatogram mass spectrum; (**b**) low energy of mass spectrum; (**c**) high energy of mass spectrum.

**Figure 8:**
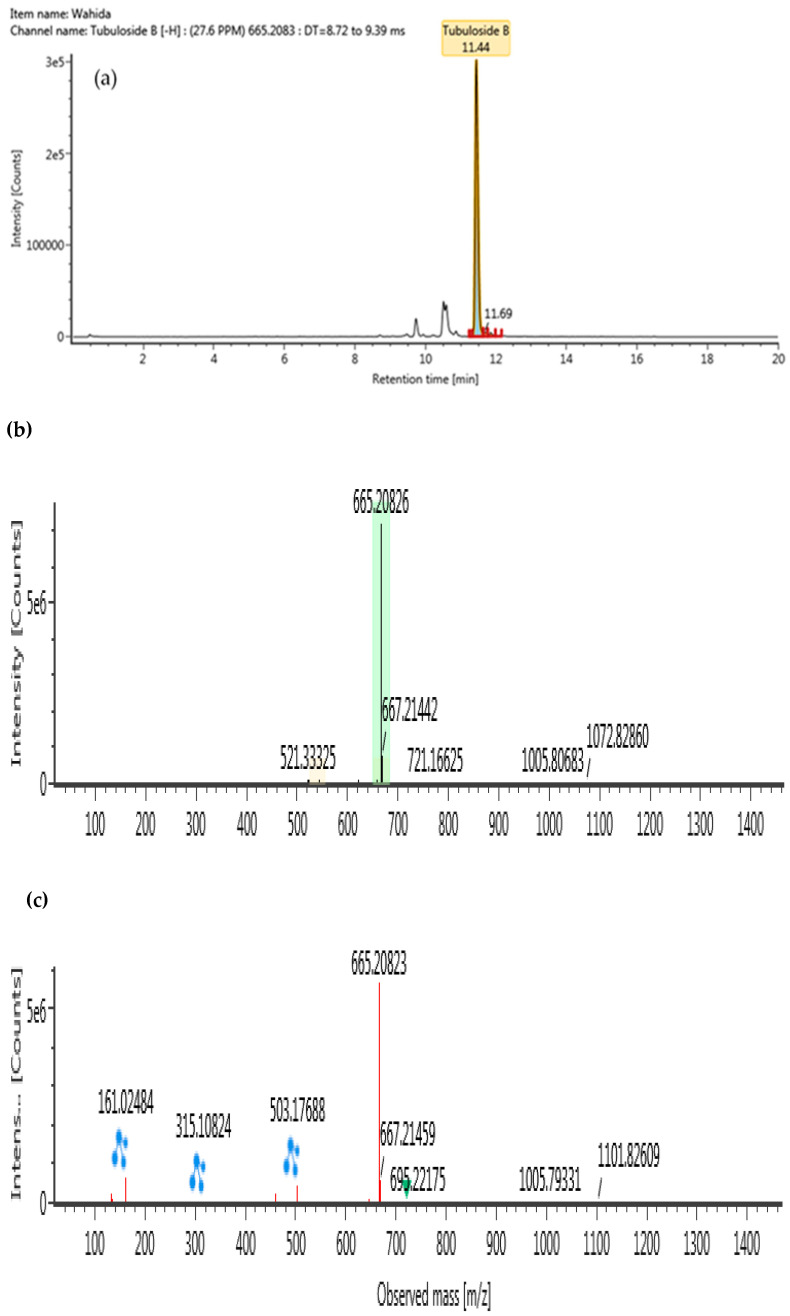
UHPLC-QTOF/MS chromatograms of tubuloside B from *Barleria lupulina* Lindl. leaf extract (BLLE): (**a**) chromatogram mass spectrum; (**b**) low energy of mass spectrum; (**c**) high energy of mass spectrum.

**Figure 9:**
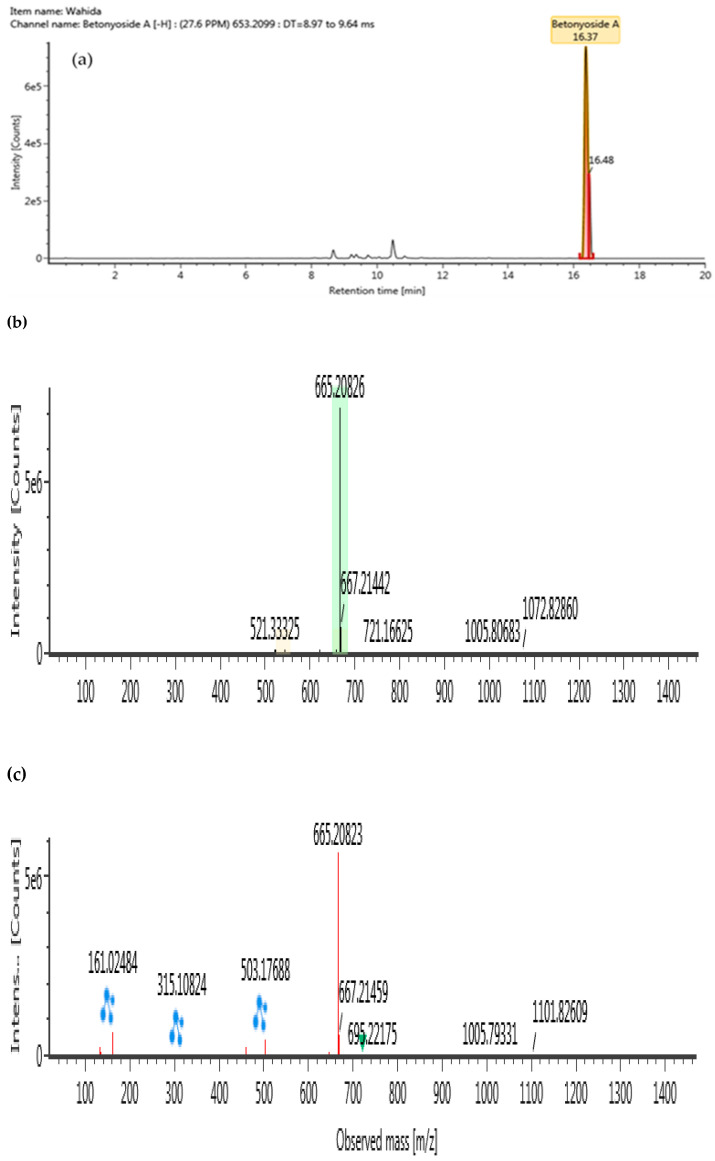
UHPLC-QTOF/MS chromatograms of betonyoside A from *Barleria lupulina* Lindl. leaf extract (BLLE): (**a**) chromatogram mass spectrum; (**b**) low energy of mass spectrum; (**c**) high energy of mass spectrum.

The ultra-high performance liquid–chromatography coupled with quadrupole time-of-flight mass spectrometry (UHPLC-QTOF/MS) was conducted in a negative ionization mode to characterize compounds from BLLE, as presented in Table 4. Using high resolution MS data, molecular formulae of all detected BLLE constituents could be assigned. Subsequent MS/MS measurements yielded accurate mass information on fragment ions and allowed identification of selected compounds by comparison with authentic standards or available literature data within the chromatograms. Following Waters library, compounds in the BLLE were tentatively identified on the basis of acquisition mass accuracy of less than 5 ppm with a theoretical fragment of more than one ion [39]. A total of four compounds were tentatively identified: lavandulifolioside, cistanoside C, tubuloside B, and betonyoside A. These newly detected compounds from BLLE were classified under phenylethanoid glycosides (PhGs). MS/MS Spectra of these compounds obtained at low and high collision energy are shown in Figure 6, Figure 7, Figure 8 and Figure 9.

The literature has stated that PhGs are generally water-soluble phenolic compounds reported to exist mostly in the families of Acanthaceae, Berberidaceae, Lamiaceae, Loganiaceae, Magnoliaceae, etc. [60]. In the present study, ethanol was selected because of its safety of use, and it is less toxic than other solvents, such as methanol and acetone. This polar organic solvent is among solvents that are typically effective in extracting PhGs from plants, especially from medicinal plants [60,61]. Several studies have shown that PhGs possess potent antioxidant activity and gives several health benefits, including antiaging, antibacterial, anticancer, antidiabetic, anti-inflammatory, antiviral, and neuroprotective properties [62,63].

## 3. Materials and Methods

### 3.1. Samples

Fresh leaves of *B. lupulina* Lindl. were collected from Serdang, Selangor, Malaysia. The species was identified by a botanist at the Herbarium of the Laboratory of Natural Products, Institute of Bioscience, Universiti Putra Malaysia, based on a voucher specimen (MFI 0047/19). Leaf samples were washed with tap water, dried in an oven at 40 °C, milled into powder, and stored in airtight containers at 4 °C for subsequent use.

### 3.2. Microwave-Assisted Extraction (MAE)

A household microwave oven (Sharp Model R202ZS, Malaysia) equipped with timing and a microwave power linearly adjustable from 200 to 1000 W was used for optimizing MAE conditions. Leaf samples (1 g) were immersed in different ethanol concentrations at a sample: solvent ratio of 1: 10 (*w*/*v*), and only one vessel was placed in the microwave oven in each experiment. The extraction was conducted in sealed vessels in 10-second interval times with no evaporation observed [36,42]. The extracts were filtered through Whatman filter paper and vacuum-dried in a rotary evaporator until the solvent was completely removed. The extracts were kept in airtight amber bottles and stored at 4 °C prior to subsequent analyses.

### 3.3. Determination of Total Phenolic Content, Total Flavonoid Content, DPPH, and ABTS Assay 

The dried extract (2 mg) was dissolved in 1 mL of the same solvent used for the extraction and subsequently used for total phenolic, total flavonoid content, and antioxidant activities.

#### 3.3.1. Total Phenolic Content

Total phenolic content (TPC) was determined based on a colorimetric method [64]. Gallic acid was used as a standard; thus, the results were expressed as the mg gallic acid equivalent (mg GAE/g) of the extracted sample. 

#### 3.3.2. Total Flavonoid Content

Total flavonoid content (TFC) was evaluated based on the procedures of [65], with some modifications. Quercetin was used as the reference standard, and the result was expressed in terms of quercetin equivalent, QE (mg of quercetin/g of extract).

#### 3.3.3. DPPH Assay

An ethanolic solution of 1-diphenyl-2-picrylhydrazyl (DPPH) radical was mixed with extract to determine antioxidant activity according to the method by [66], with some modifications. After 30 minutes of incubation, the mixture of the extract and DPPH was measured by using a UV–VIS microplate reader at 515 nm. Radical scavenging activity was expressed as the inhibition percentage and was calculated using the following formula (5):

% Inhibition = ((A control − A sample))/A control × 100(5)

#### 3.3.4. ABTS Assay

The ABTS assay was conducted to determine the antioxidant activity of BLLE against ABTS radicals, with some modifications [67]. An ethanolic of radical solution (7 mM ABTS and 2.45 mM potassium persulfate (K_2_S_2_O_8_)) was kept in the dark at room temperature for 12–16 h prior to use. The radical solution was diluted with ethanol to obtain an absorbance of 0.70 ± 0.02 at 734 nm. The solution was mixed with the extract of interest and allowed to stand in the dark for 15 min before reading at 734 nm. The radical scavenging activity was expressed as the inhibition percentage and was calculated using the following formula (6):

% Inhibition = ((A control-A sample ))/A control × 100(6)

### 3.4. Experimental Design and Statistical Analysis

#### 3.4.1. Single-Factor Analysis

The effects of the three independent variables, including ethanol concentrations (20–95%, *v/v*), extraction time (30–150 s), and microwave power (200–1000 W), were selected based on previous studies [29,30]. Single-factor analysis was used to investigate the effects of the variables on TPC, TFC, DPPH, and ABTS. 

#### 3.4.2. Response Surface Methodology (RSM)

Based on single-factor analysis, RSM was applied by using Design Expert software (Version 10) to construct a Box–Behnken design (BBD) with five central-point replicates to determine the effects of variables on responses. Three independent variables, namely ethanol concentrations (A), microwave power (B), extraction time (C), and their values and levels, are presented in Table 5. Four responses (TPC, TFC, DPPH, and ABTS) were chosen for a total of 17 experiments (Table 6).

### 3.5. Identification of Compounds

Ultra-high-performance liquid–chromatography (UHPLC-QTOF/MS) and a Waters Acquity ultra-performance LC system (Waters, Milford, MA, USA) were used in identifying bioactive compounds. A column (ACQUITY UPLC HSS T3, 100 mm × 2.1 mm × 1.8 µm, Waters, Manchester, UK) was used to separate the chromatographic compounds. A linear binary gradient was used for mobile phases A (0.1% formic acid) and B (acetonitrile) followed by a multistep gradient: 0 min, 1% B and 99% A; 0.5 min, 1% B and 99% A; 16.00 min, 35% B and 65% A; 18.00 min, 100% B and 0% A; and 20.00 min, 1% B and 99% A. An aliquot of 1 µL injection volume of the sample at 0.6 mL/min flow rate was set. Data were acquired in an independent data analysis (IDA) in the range *m/z* of 50–1500 at 0.1 s/scan in high-definition mass spectrometry elevated energy (HDMSE) mode with two independent scans and different collision energies (CE). The run of a low-energy (LE) scan was at a fixed CE of 4 eV, and in a high-energy (HE) scan, the CE was ramped from 10 to 40 eV.

### 3.6. Statistical Analysis

One-way analysis of variance (ANOVA, SAS 9.4, SAS Institute Inc, Cary, NC, USA) was used to calculate the difference in means among different parameters in the single-factor analysis *(p* < 0.05). The optimal values of the three response variables were predicted by their optimal value by constructing a BBD using Design Expert 10 software. All experimental results were carried out in triplicates, and results are reported as means ± SD.

## 4. Conclusions

The conditions for optimal extraction of *B. lupulina* Lindl. leaf extracts were investigated using microwave-assisted extraction procedures. Three independent variables (ethanol concentration, microwave power, and extraction time) that give an optimum value of TPC, TFC, and antioxidant activities obtained from single-factor experiments were further optimized by response surface methodology (RSM) based on a Box–Behnken design. The quadratic models obtained by RSM were accurate and reliable in which R^2^ and adjusted R^2^ were more than 0.90 with a non-significant lack of fit at *p* > 0.05. The optimal extraction conditions showed 80% (*v/v*) of ethanol concentration with a microwave power of 400 W, and an extraction time of 30 s resulted in a 238.71 mg gallic acid equivalent (GAE)/g sample (TPC), 58.09 mg QE/g sample (TFC), 87.95% (DPPH), and 89.56% (ABTS). Results from the validation experiments are in agreement with predicted values. The UHPLC-QTOF-MS confirmed four new phenylethanoid glycosides compounds in BLLE. We suggest that the optimal conditions of the extract be further studied in in vivo antioxidant activity.

## Figures and Tables

**Table 1:** Analysis of variance for the fitted model of experiment.

		TPC	TFC	DPPH	ABTS
Model	F-Value	20.17	23.52	350.98	29.12
	*p*-Value	0.0003	0.0002	<0.0001	<0.0001
Lack of fit		0.7414	0.8221	0.5152	0.49447
R^2^		0.9629	0.9680	0.9978	0.9740
Adjusted R^2^		0.9151	0.9268	0.9949	0.9405

TPC: total phenolic content; TFC: total flavonoid content; DPPH: 1-diphenyl-2-picrylhydrazyl; ABTS: 2,20-azino-bis (3-ethylbenzothizoline-6-sulfonic acid).

**Table 2:** Analysis of variance of experimental results.

Parameter		TPC		TFC		DPPH		ABTS	
	df	Estimated Coefficients	Prob > F	Estimated Coefficients	Prob > F	Estimated Coefficients	Prob > F	Estimated Coefficients	Prob > F
Linear									
A	1	32.90	<0.0001	10.97	<0.0001	4.95	<0.0001	21.78	<0.0001
B	1	−8.23	0.0225	−4.11	0.0040	−0.11	0.4225	−5.60	0.0112
C	1	−6.79	0.0471	−1.80	0.1088	−0.72	0.0007	−4.44	0.0302
Quadratic									
A^2^	1	−13.57	0.0102	7.95	0.0006	−4.85	<0.0001	−12.75	0.0008
B^2^	1	−3.74	0.3689	−2.95	0.0650	−1.39	<0.0001	4.69	0.0760
C^2^	1	1.64	0.6867	1.09	0.4452	3.05	<0.0001	2.92	0.2371
Interaction									
AB	1	9.54	0.0482	3.17	0.0556	3.82	<0.0001	−6.47	0.0267
AC	1	4.42	0.3053	5.91	0.0037	0.65	0.0081	−9.74	0.0040
BC	1	-13.33	0.0124	−1.72	0.2535	−0.42	0.0519	−4.71	0.0812

df: degree of freedom; TPC: total phenolic content; TFC: total flavonoid content; DPPH: 1-diphenyl-2-picrylhydrazyl (antioxidant activity); ABTS: 2,20-azino-bis (3-ethylbenzothizoline-6-sulfonic acid) (anti-oxidant activity); A: ethanol concentration; B: microwave power; C: extraction time.

**Table 3:** Predicted and experimental values of responses.

	TPC (mg GAE/g Sample)	TFC (mg QE/g Sample)	DPPH (%)	ABTS (%)
Predicted	239.77	58.27	88.62	90.29
Experimental	238.71	58.09	87.95	89.56

TPC: total phenolic content; TFC: total flavonoid content; DPPH: 1-diphenyl-2-picrylhydrazyl (antioxidant activity); ABTS: 2,20-azino-bis (3-ethylbenzothizoline-6-sulfonic acid) (antioxidant activity).

**Table 4:** Identification of phenylethanoid glycosides compounds in the *Barleria lupulina* Lindl. leaf extract with MS/MS fragments.

Compound Name	Formula	Ion	Natural Mass (Da)	Observed *m*/*z*	∆ppm	Retention Time (min)	Ion Fragments
Lavandulifolioside	C_34_H_44_O_19_	M-H	756.24768	755.2423	2.5	8.54	695.21791; 711.24631
Cistanoside C	C_30_H_38_O_15_	M-H	638.22107	637.2137	−0.1	10.28	160.01726; 319.13008; 461.16654
Tubuloside B	C_31_H_38_O_16_	M-H	666.21599	665.2083	−0.7	11.44	161.02484; 315.10824; 503.17688
Betonyoside A	C_30_H_38_O_16_	M-H	654.21599	653.2099	1.9	16.37	161.02484; 315.10824; 503.17688

**Table 5:** Independent variable for the Box–Behnken design.

Independent Variable	Label	Levels		
		−1	0	1
Ethanol concentration (%, *v/v*)	A	20	50	80
Microwave Power (W)	B	200	400	600
Extraction time (s)	C	30	75	120

**Table 6:** Box–Behnken design with responses of variables.

	Factors			Responses			
	A	B	C	1	2	3	4
Run	Ethanol Concentration	Microwave Power	Irradiation Time	TPC	TFC	DPPH	ABTS
	%, *v/v*	W	s	mg GAE/g	mg QE/g	%	%
1	50	200	120	233.33	45.82	87.07	78.48
2	50	400	75	210.00	40.92	85.61	64.79
3	20	400	30	186.50	49.38	80.30	30.08
4	20	200	75	179.33	42.34	78.08	34.42
5	80	200	75	230.17	59.26	80.47	91.96
6	50	200	30	217.00	44.81	87.27	73.30
7	80	400	30	239.33	58.19	88.75	92.08
8	80	400	120	231.33	65.27	88.22	59.11
9	20	600	75	149.00	29.15	70.21	33.20
10	50	400	75	227.17	44.58	84.87	66.87
11	50	400	75	224.17	46.68	85.24	59.25
12	50	600	120	185.00	32.77	86.03	60.83
13	50	400	75	215.33	41.42	85.81	70.16
14	50	400	75	205.50	38.24	85.52	59.75
15	80	600	75	238.00	58.75	87.89	64.86
16	50	600	30	222.00	38.64	87.90	74.50
17	20	400	120	160.83	32.80	77.16	36.05
